# The epidemiology of minor surgical problems during specialists' absence: Single center, descriptive study

**DOI:** 10.1002/jgf2.337

**Published:** 2020-06-30

**Authors:** Toshiyuki Abe, Tomoyasu Matsubara, Sho Sasaki, Hiroyuki Oda, Hiroshi Imura, Tsunetoshi Mogi

**Affiliations:** ^1^ Department of General Internal Medicine Iizuka Hospital Iizuka Japan; ^2^ Department of Gastroenterology Iizuka Hospital Iizuka Japan; ^3^ Department of Clinical Neuroscience and Therapeutics Hiroshima University Graduate School of Biomedical and Health Sciences Hiroshima Japan; ^4^ Department of Nephrology/Clinical Research Support Office Iizuka Hospital Iizuka Japan; ^5^ Department of Healthcare Epidemiology Kyoto University Graduate School of Public Health Kyoto Japan; ^6^ Department of General and Family Medicine Kurume University Medical Center Kurume Japan

**Keywords:** emergency medicine, epidemiology, family medicine, minor surgical procedures, off‐hours care

## Abstract

**Background:**

In Japan, even if physicians have not experienced surgical training, they face many instances in which they must provide initial surgical treatment, especially during off‐hours. This study aimed to identify the frequency and fields of commonly encountered problems in a Japanese emergency department.

**Methods:**

A retrospective review was performed to identify walk‐in outpatients with exogenous problems visiting during off‐hours in the Japanese educational hospital providing primary to tertiary emergency care between January 1 and December 31, 2014. Diseases were aggregated according to International Classification of Primary Care (Second Edition; ICPC‐2).

**Results:**

During the study period, 33 424 patients visited and 7476 were classified into the “exogenous” group. We analyzed the data of 7421 patients after excluding 55 who were deemed undiagnosable based on reviews of the charts. The median age of patients who visited the ED during off‐hours was 29 years (range: 0‐101 years, IQR: 8‐60 years). Altogether, 226 types of problems included in ICPC‐2 were identified during the study period. The majority fields of exogenous problems were ‘skin,’ ‘Musculoskeletal,’ and ‘eye.’ The 15 problems with the highest frequencies accounted for 50.2% of the total problems.

**Conclusions:**

We identified surgical problems with high treatment frequencies among patients visiting the ED during off‐hours. Providing education focusing on these frequent surgical problems can help to improve the initial treatment quality and reduce the anxiety for those doctors who provide initial surgical treatment.

## Graphical abstract

We identified surgical diseases with high treatment frequencies among patients during off‐hours. The findings can help to develop more effective training programs based on frequency of problem.

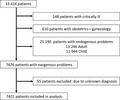

## 1 INTRODUCTION

Minor surgical problems that are nonfatal and immediately handled in the emergency department (ED) or outpatient clinic are common issues in clinical settings. Ideally, specialists in each department should be prepared and available for treatment; however, owing to human resource constraints, this is not always the case. Emergency doctors possess the necessary expertise to handle unexpected situations of various kinds; however, because of their regional disparities, the number of emergency doctors in Japan is insufficient.^1^

Therefore, in Japan, physicians who have never experienced surgical training are required to provide initial treatment for surgical problems. In such a context, it is useful for these doctors to understand which minor surgical problems are most frequently encountered in the ED in order to prioritize the areas that they should learn about.

Several studies have reported field‐wise disease frequencies in outpatient departments and EDs.^2^, ^3^, ^4^, ^5^, ^6^ However, there have been few epidemiological studies regarding surgical problems across different specialties when specialists were not available, namely during off‐hours.^7^ In addition, the types of presenting problems also vary, depending on whether treatment occurs at specialized outpatient departments or within/outside of practice hours.^5^, ^8^ Additionally, there are other factors with potential variations across countries, including access to medical institutions and differing insurance systems,^2^ especially for minor conditions. Therefore, epidemiological studies regarding minor surgical problems during off‐hours in Japan are required. This study aimed to identify the frequency and fields of commonly encountered problems in a Japanese ED.

## 2 METHODS

### 2.1 Study design and settings

We conducted a descriptive study through a retrospective review of patients who attended our outpatient department during off‐hours between January 1 and December 31, 2014. Our hospital is a 1000‐bed educational hospital located in a suburban area in Japan. The hospital's emergency medical center is the only one in the region, providing primary to tertiary emergency care and covering approximately 180 000 people in the medical area.^9^ Annual numbers of emergency walk‐in outpatients and ambulances accepted were approximately 30 000 and 8000, respectively.

### 2.2 Study participants

Walk‐in outpatients who visited during off‐hours (16:30‐8:30 on weekdays and all hours on weekends) were classified by trained nurses into five groups based on their complaints and vital signs: endogenous, exogenous, pediatric, obstetrics‐gynecology, and critically ill. Patients who had unstable vital signs at any time were sorted into the critically ill group. For the purpose of this study, we included only those patients who were classified as the exogenous group. Further, we excluded patients whose presenting disease was unidentifiable through a review of their medical records.

### 2.3 Measurements

The data were collected from the hospital medical records included age, gender, and clinical modification codes from the International Classification of Diseases, 10th Revision (ICD‐10). When the presenting disease was unidentifiable, two physicians (TA and TM) who are board‐certified members of the Japanese Society of Internal Medicine and were trained in primary care and emergency medicine examined the medical charts.

Subsequently, the ICD‐10 codes were transferred to the corresponding International Classification of Primary Care (Second Edition; ICPC‐2) codes using the ICPC‐2 comparison table (electronic version 7.0).^10^, ^11^ The ICD‐10 involves comprehensive classifications combining lesion site and etiology; however, this was too detailed for the present study and there was the possibility that what we considered individual problems may have been separated into numerous minor conditions in the ICD‐10. Therefore, we decided to convert the diagnosis to ICPC‐2, which uses general disease or problem names.

We aimed to uncover the frequency and disease composition of minor surgical problems in patients who attended the outpatient department during off‐hours. In addition, we examined whether the disease frequency changed with age. Therefore, we also divided the patients into three age subgroups: children (0–14 years old), adults (15–64 years old), and elderly (65 years and older).

### 2.4 Statistical analysis and ethics

Comparison between the ages of the participants using the ICPC‐2 field was performed using a chi‐square test or Fisher's exact test. A 2‐sided *P*‐value < .05 was considered statistically significant. Numerical variables were expressed as median, range, and interquartile range (IQR), and categorical variables were expressed as number and percentage. The data were analyzed using STATA 14.0 (StataCorp).

The ethics committee approved this study (approval no. 15 194). This study was conducted in accordance with the principles of the Declaration of Helsinki.^12^ This manuscript was written according to the STROBE (STrengthening the Reporting of OBservational studies in Epidemiology) guidelines.^13^

## 3 RESULTS

**FIGURE 1:**
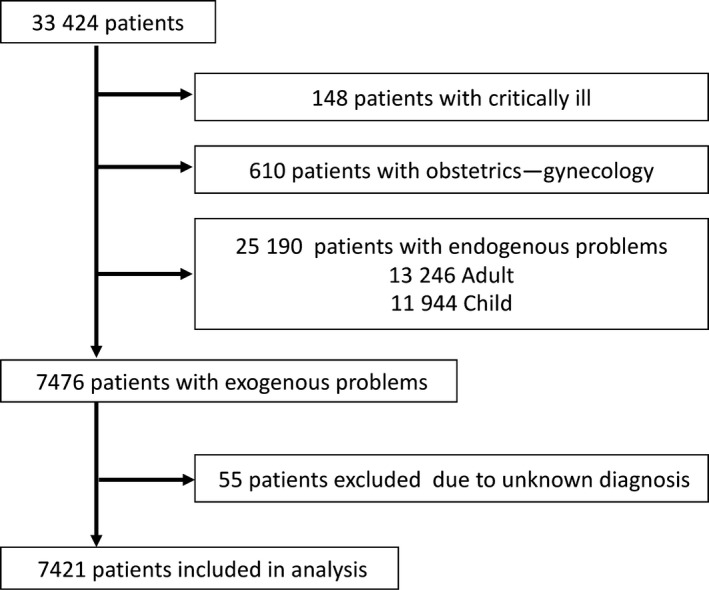
Study flow

During the study period, 33 424 patients visited the ED during off‐hours; of these, 7476 were classified into the exogenous group. We analyzed the data of 7421 patients after excluding 55 who were deemed undiagnosable after reviewing the charts (Figure 1).

The median age of patients who visited the ED during off‐hours was 29 years (range: 0‐101 years, IQR: 8‐60 years), and 55.3% (n = 4102) of the patients were male, while 44.7% (n = 3319) were female. Of the patients classified as exogenous, 424 patients (3.6%) were given a diagnosis for their most probable disease, as a definitive diagnosis was not made in the ED. Altogether, 226 types of problems included in the ICPC‐2 were identified during the study period.

The majority of the patients had injuries to Skin (S, 38.3%), Musculoskeletal (L, 31.6%), or Eye (F, 8.6%). ICPC‐2‐coded disease fields are presented in Table 1. In addition, the differences of the disease frequency were observed between age‐groups (children, adults, and elderly) in S, L, and F as well as the General and unspecified (A), Ear (H), Circulatory (K), Neurological (N), Respiratory (R), Endocrine, metabolic, and nutritional (T), and Urological (U) fields as shown Table 1.

**Table 1:** Distribution of ICPC‐2 field, gender, and ages

ICPC‐2 field	Children	Adults	Elderly	*P*‐value	Grand total number (%)
	Number (%)	Number (%)	Number (%)		
A [General and unspecified]	146 (5.7)	309 (9.4)	143 (9.5)	<.05*	598 (8.1)
B [Blood, blood‐forming organs, and immune mechanism]	0 (0.0)	3 (0.1)	2 (0.1)	.18**	5 (0.1)
D [Digestive]	94 (3.6)	94 (2.8)	60 (4.0)	.06*	248 (3.3)
F [Eye]	252 (9.6)	291 (8.8)	98 (6.5)	<.05*	641 (8.6)
H [Ear]	55 (2.1)	64 (1.9)	15 (1.0)	<.05*	134 (1.8)
K [Circulatory]	3 (0.1)	17 (0.5)	16 (1.1)	<.05*	36 (0.5)
L [Musculoskeletal]	657 (26.0)	1218 (36.4)	473 (31.4)	<.05*	2348 (31.6)
N [Neurological]	65 (2.5)	40 (1.1)	45 (3.0)	<.05*	150 (2.0)
P [Psychological]	2 (0.1)	13 (0.4)	5 (0.3)	.06*	20 (0.3)
R [Respiratory]	101 (3.8)	100 (3.1)	88 (5.9)	<.05*	289 (3.9)
S [Skin]	1194 (45.7)	1122 (33.7)	528 (35.1)	<.05*	2844 (38.3)
T [Endocrine, metabolic, and nutritional]	0 (0.0)	21 (0.6)	14 (0.9)	<.05**	35 (0.5)
U [Urological]	2 (0.1)	13 (0.4)	8 (0.5)	<.05*	23 (0.3)
W [Pregnancy, childbearing, family planning]	1 (0.0)	1 (0.0)	0 (0.0)	.76**	2 (0.0)
X [Female genital system]	8 (0.3)	8 (0.2)	2 (0.1)	.54*	18 (0.2)
Y [Male genital system]	10 (0.4)	12 (0.3)	7 (0.5)	.86*	29 (0.4)
Z [Social problems]	0 (0.0)	1 (0.0)	0 (0.0)	.54**	1 (0.0)
Total	2590 (100)	3327 (100)	1504 (100)		7421 (100)

* Chi‐square test.

** Fisher's exact test.

When considering the individual disease items, the most frequent problem in the ED was [S16: Bruise/contusion] (n = 1288, 17.4%), followed by [S18: Laceration/cut] (n = 789, 10.6%), [S14: Burn/scald] (n = 227, 3.1%), [L74: Fracture: hand/foot bone] (n = 219, 3.0%), and [L72: Fracture: radius/ulna] (n = 166, 2.2%). The 25 surgical problems with the highest frequencies, excluding “other,” are shown in Table 2. The 15 items with the highest frequencies accounted for 50.2% of the total.

**Table 2:** Top 25 frequent ICPC‐2 codes of surgical problems of all ages

No.	ICPC‐2 code	ICPC‐2E title	Number of patients	Ratio (%)	Cumulative ratio (%)
1	S16	Bruise/contusion	1225	16.5	16.5
2	S18	Laceration/cut	789	10.6	27.1
3	S14	Burn/scald	227	3.1	30.2
4	L74	Fracture: hand/foot bone	219	3.0	33.1
5	L72	Fracture: radius/ulna	166	2.2	35.4
6	L03	Low back symptom/complaint	145	2.0	37.3
7	S13	Animal/human bite	141	1.9	39.2
8	R06	Nose bleed/epistaxis	139	1.9	41.1
9	L77	Sprain/strain of ankle	122	1.6	42.8
10	F75	Contusion/hemorrhage eye	117	1.6	44.3
11	A81	Multiple trauma/injuries	117	1.6	45.9
12	R87	Foreign body nose/larynx/bronchus	89	1.2	47.1
13	S17	Abrasion/scratch/blister	89	1.2	48.3
14	F70	Conjunctivitis infectious	72	1.0	49.3
15	D82	Teeth/gum disease	68	0.9	50.2
16	L73	Fracture: tibia/fibula	68	0.9	51.1
17	F76	Foreign body in eye	60	0.8	51.9
18	L80	Dislocation/subluxation	60	0.8	52.7
19	S15	Foreign body in skin	53	0.7	53.4
20	L18	Muscle pain	47	0.6	54.1
21	L15	Knee symptom/complaint	46	0.6	54.7
22	A87	Complication of medical treatment	44	0.6	55.3
23	D79	Foreign body digestive system	43	0.6	55.9
24	L75	Fracture: femur	43	0.6	56.4
25	S88	Dermatitis contact/allergic	38	0.5	57.0

Note

The results are rounded to one decimal place.

Abbreviation: ICPC‐2, International Classification of Primary Care, Second Edition.

The disease compositions and frequencies for each age‐group are shown in Table 3. The 9, 18, and 13 items accounted for over 50% of the subgroup total in the children, adults, and elderly group, respectively.

**Table 3:** Top 25 frequent ICPC‐2 codes of surgical problems of children, adults, and elderly

No.	Children (0‐14 y)				Adults (15‐64 y)				Elderly (65‐101 y)			
	ICPC‐2 code	ICPC‐2E title	Number of patients (%)	Cumulative ratio (%)	ICPC‐2 code	ICPC‐2E title	Number of patients (%)	Cumulative ratio (%)	ICPC‐2 code	ICPC‐2E title	Number of patients (%)	Cumulative ratio (%)
1	S16	Bruise/contusion	640 (24.7)	24.7	S18	Laceration/cut	401 (12.1)	12.1	S16	Bruise/contusion	247 (16.4)	16.4
2	S18	Laceration/cut	250 (9.7)	34.4	S16	Bruise/contusion	338 (10.2)	22.2	S18	Laceration/cut	138 (9.2)	25.6
3	S14	Burn/scald	130 (5.0)	39.4	L74	Fracture: hand/foot bone	149 (4.5)	26.7	R06	Nose bleed/epistaxis	62 (4.1)	29.7
4	F75	Contusion/hemorrhage eye	65 (2.5)	41.9	L03	Low back symptom/complaint	83 (2.5)	29.2	L03	Low back symptom/complaint	56 (3.7)	33.4
5	L72	Fracture: radius/ulna	53 (2.0)	43.9	S14	Burn/scald	82 (2.5)	31.7	L72	Fracture: radius/ulna	50 (3.3)	36.8
6	R87	Foreign body nose/larynx/bronchus	52 (2.0)	45.9	L77	Sprain/strain of ankle	80 (2.4)	34.1	L75	Fracture: femur	37 (2.5)	39.2
7	L74	Fracture: hand/foot bone	46 (1.8)	47.7	S13	Animal/human bite	80 (2.4)	36.5	A81	Multiple trauma/injuries	33 (2.2)	41.4
8	S17	Abrasion/scratch/blister	36 (1.4)	49.1	L72	Fracture: radius/ulna	63 (1.9)	38.4	S13	Animal/human bite	30 (2.0)	43.4
9	S15	Foreign body in skin	34 (1.3)	50.4	R06	Nose bleed/epistaxis	55 (1.7)	40.0	S17	Abrasion/scratch/blister	25 (1.7)	45.1
10	L77	Sprain/strain of ankle	33 (1.3)	51.7	A81	Multiple trauma/injuries	52 (1.6)	41.6	L74	Fracture: hand/foot bone	24 (1.6)	46.7
11	A81	Multiple trauma/injuries	32 (1.2)	52.9	F70	Conjunctivitis infectious	50 (1.5)	43.1	F75	Contusion/hemorrhage eye	24 (1.6)	48.3
12	S13	Animal/human bite	31 (1.2)	54.1	F76	Foreign body in eye	41 (1.2)	44.3	R87	Foreign body nose/larynx/bronchus	18 (1.2)	49.5
13	R06	Nose bleed/epistaxis	22 (0.8)	55.0	L80	Dislocation/subluxation	39 (1.2)	45.5	D82	Teeth/gum disease	16 (1.1)	50.5
14	D79	Foreign body digestive system	19 (0.7)	55.7	L73	Fracture: tibia/fibula	37 (1.1)	46.6	S14	Burn/scald	15 (1.0)	51.5
15	F70	Conjunctivitis infectious	18 (0.7)	56.4	D82	Teeth/gum disease	35 (1.1)	47.6	L73	Fracture: tibia/fibula	14 (0.9)	52.5
16	L73	Fracture: tibia/fibula	17 (0.7)	57.1	A87	Complication of medical treatment	32 (1.0)	48.6	D79	Foreign body digestive system	14 (0.9)	53.4
17	D82	Teeth/gum disease	17 (0.7)	57.7	L86^a^	Back syndrome with radiating pain	30 (0.9)	49.5	L15	Knee symptom/complaint	13 (0.9)	54.3
18	S22^a^	Nail symptom/complaint	16 (0.6)	58.3	L18	Muscle pain	29 (0.9)	50.4	L80	Dislocation/subluxation	12 (0.8)	55.1
19	N79^a^	Concussion	15 (0.6)	58.9	F75	Contusion/hemorrhage eye	28 (0.8)	51.2	S88	Dermatitis contact/allergic	12 (0.8)	55.9
20	L12^a^	Hand/finger symptom/complaint	14 (0.5)	59.5	S17	Abrasion/scratch/blister	28 (0.8)	52.1	L18	Muscle pain	11 (0.7)	56.6
21	H74^a^	Chronic otitis media	10 (0.4)	59.8	L15	Knee symptom/complaint	26 (0.8)	52.8	A87	Complication of medical treatment	11 (0.7)	57.3
22	L80	Dislocation/subluxation	9 (0.3)	60.2	S88	Dermatitis contact/allergic	20 (0.6)	53.4	F76	Foreign body in eye	11 (0.7)	58.0
23	L09^a^	Arm symptom/complaint	9 (0.3)	60.5	L83^a^	Neck syndrome	19 (0.6)	54.0	L77	Sprain/strain of ankle	9 (0.6)	58.6
24	L78^a^	Sprain/strain of knee	8 (0.3)	60.8	R87	Foreign body nose/larynx/bronchus	19 (0.6)	54.6	L92^a^	Shoulder syndrome	8 (0.5)	59.2
25	S04^a^	Lump/swelling localized	8 (0.3)	61.2	F72^a^	Blepharitis/stye/chalazion	18 (0.5)	55.1	L14^a^	Leg/thigh symptom/complaint	7 (0.5)	59.6

^a^ Not included in the overall top 25.

## 4 DISCUSSION

This study examined the types and frequencies of minor surgical problems treated at an ED during off‐hours in Japan. Of the patients who visited the ED during off‐hours, 22% had surgical problems; specifically, the problems with the highest frequencies were those related to the skin (ICPC‐2 code “S”: 38.3%), musculoskeletal (ICPC‐2 code “L”: 31.6%), and eye (ICPC‐2 code “F”: 8.6%). A Greek study has also shown high treatment frequencies for these fields.^7^ After adding patients with epistaxis (ICPC‐2 classified the respiratory field) to the ear field (ICPC‐2 code H), the indication corresponded with otorhinolaryngology problems, accounting for high frequencies in our study: n = 273, 3.6% of all patients. After this correction, the disease composition was in line with the Greek study. Thus, although there is a limitation that few studies were reported, trends in frequently encountered disease fields in the ED appear to be similar across countries.

The idea that the disease frequency varied with age was also confirmed. Differences of the disease frequency between age‐groups were observed in 10 out of 17 fields of ICPC‐2. This result suggests the importance of the age‐appropriate disease frequency, although, as discussed below, we should consider the frequency not only by fields but also by each disease.

The 15 most frequent problems included in the ICPC‐2 codes and excluding “other” cases accounted for 50.2% of the surgical problems treated in the ED during off‐hours. Given that 226 types of problems included in ICPC‐2 were encountered over the entire study period, these 15 problems accounted for a large proportion of the surgical problems. Further examination of the age subgroups revealed that 9 and 13 items accounted for over 50% of the total, in children and the elderly group, respectively. Although the disease frequencies were somewhat varied, the disease composition of the top‐ranking diseases shared many items across all groups. These results suggest that there was a specific trend of disease frequencies encountered during off‐hours; these 15 problems can be leverage points. In addition, the initial treatment for these 15 problems could include procedures that do not require a high degree of expertise. Thus, although it is necessary to consider whether the prognosis of these problems can be improved through treatment intervention in 15 problems, the results underscore the fact that the doctors treating surgical problems in the ED should prioritize the acquisition of knowledge about these truly common surgical problems as well as treatment for critical conditions.

Presenting disease compositions in the ED differed from those in the daytime outpatient department. A Japanese epidemiological study of a dermatology department identified high frequencies of atopic dermatitis, psoriasis, and a fungal infection: *tinea unguium*;^5^ but these diseases were uncommon during EDs' off‐hours in our study. Diseases in other fields also varied in frequency, depending on whether the data came from daytime specialists' outpatient departments or EDs.

This study had several limitations. We only studied patients classified as having surgical problems by triage nurses; thus, it is possible that patients were erroneously sorted. Further, it is challenging to differentiate whether some problems (eg, inflammatory ear disease *otitis media* and hives) are endogenous, exogenous, or pediatric. Although diagnoses were made to provide initial treatment, definitive diagnoses were not recorded for all patients. Nonetheless, our results can assist clinicians in identifying the frequency of minor surgical problems in the ED. Finally, as outcome evaluations were not performed, it is unclear whether training based on disease frequency obtained from this study will improve patients' outcomes.

## 5 CONCLUSION

We identified problems with high treatment frequencies among patients with surgical problems who visited the ED during off‐hours. If doctors improve their knowledge about these problems, it may be possible to improve the initial treatment quality.

Providing education focusing on these frequent surgical problems may help to improve the initial treatment quality and reduce the anxiety of the doctors who provide initial surgical treatment.

## 6 ACKNOWLEDGEMENTS

None.

## 7 CONFLICT OF INTEREST

The authors have stated explicitly that there are no conflicts of interest in connection with this article.

## References

[1] Mizuno J , Hanaoka K . Regional differences in the numbers of Emergency Medical Doctors and Emergency Medical Institutions in Japan. J Japanese Assoc Acute Med. 2004;15(11):593–604.

[2] Channa R , Zafar SN , Canner JK , Haring RS , Schneider EB , Friedman DS . Epidemiology of eye‐related emergency department visits. JAMA Ophthalmol. 2016;134(3):312–9. 26821577 10.1001/jamaophthalmol.2015.5778

[3] Kozin ED , Sethi RK , Remenschneider AK , Kaplan AB , Del Portal DA , Gray ST , et al. Epidemiology of otologic diagnoses in United States emergency departments. Laryngoscope. 2015;125(8):1926–33. 25702897 10.1002/lary.25197 PMC4512842

[4] Baibergenova A , Shear NH . Skin conditions that bring patients to emergency departments. Arch Dermatol. 2011;147(1):118–20. 20855676 10.1001/archdermatol.2010.246

[5] Furue M , Yamazaki S , Jimbow K , Tsuchida T , Amagai M , Tanaka T , et al. Prevalence of dermatological disorders in Japan: a nationwide, cross‐sectional, seasonal, multicenter, hospital‐based study. J Dermatol. 2011;38(4):310–20. 21426384 10.1111/j.1346-8138.2011.01209.x

[6] Marinos G , Giannopoulos A , Vlasis K , et al. Primary care in the management of common orthopaedic problems. Qual Prim Care. 2008;16(5):345–9. (in eng). 18973716

[7] Marinos G , Vasileiou I , Katsargyris A , Klonaris CP , Korombelis P , Michail O , et al. Management of minor medical problems and trauma: the role of general practice. Rural Remote Health. 2009;9(4):1019. 19883145

[8] Izumida H , Sasaki J , Tajima K , Suzuji M , Fujishima S , Ogawa K , et al. Epistaxis cared by emergency physicians in emergency department. J Japanese Assoc Acute Med. 2014;25:93–101.

[9] Japan Medical Association . Japan Medical Analysis Platform. http://jmap.jp/. Accessed March 3, 2020.

[10] Okkes I , Jamoulle M , Lamberts H , Bentzen N . ICPC‐ 2‐E: the electronic version of ICPC‐2. Differences from the printed version and the consequences. Fam Pract. 2000;17(2):101–7. 10758069 10.1093/fampra/17.2.101

[11] Wonca International Classification Committee (WICC) . ICPC‐2e – English version. https://ehelse.no/kodeverk/icpc-2e--english-version. Accessed March 3, 2020.

[12] World Medical Association . World Medical Association Declaration of Helsinki: ethical principles for medical research involving human subjects. JAMA. 2013;310(20):2191–4. 24141714 10.1001/jama.2013.281053

[13] von Elm E , Altman DG , Egger M , Pocock SJ , Gotzsche PC , Vandenbroucke JP . The Strengthening the Reporting of Observational Studies in Epidemiology (STROBE) Statement: guidelines for reporting observational studies. Int J Surg. 2014;12(12):1495–9. 25046131 10.1016/j.ijsu.2014.07.013

